# Manual Planimetry of the Medial Temporal Lobe Versus Automated Volumetry of the Hippocampus in the Diagnosis of Alzheimer's Disease

**DOI:** 10.7759/cureus.544

**Published:** 2016-03-26

**Authors:** Manuel Menéndez González, Esther Suárez-Sanmartin, Ciara García, Pablo Martínez-Camblor, Eric Westman, Andy Simmons

**Affiliations:** 1 Neurology, Hospital Universitario Central de Asturias; 2 Morphology and Cellular Biology, Universidad de Oviedo; 3 Facultad de Ciencias de la Salud, Universidad Autónoma de Chile; 4 Estadística, Hospital Universitario Central de Asturias; 5 Department of Neurobiology, Care Sciences and Society, Karolinska Institutet; 6 Institute of Psychiatry, King's College London

**Keywords:** mri, planimetry, volumetry, alzheimer’s disease, medial temporal lobe, hippocampus, addneuromed study

## Abstract

Introduction:

Though a disproportionate rate of atrophy in the medial temporal lobe (MTA) represents a reliable marker of Alzheimer’s disease (AD) pathology, measurement of the MTA is not currently widely used in daily clinical practice. This is mainly because the methods available to date are sophisticated and difficult to implement in clinical practice (volumetric methods), are poorly explored (linear and planimetric methods), or lack objectivity (visual rating). Here, we aimed to compare the results of a manual planimetric measure (the yearly rate of absolute atrophy of the medial temporal lobe, 2D-yrA-MTL) with the results of an automated volumetric measure (the yearly rate of atrophy of the hippocampus, 3D-yrA-H).

Methods:

A series of 1.5T MRI studies on 290 subjects in the age range of 65–85 years, including patients with AD (n = 100), mild cognitive impairment (MCI) (n = 100), and matched controls (n = 90) from the AddNeuroMed study, were examined by two independent subgroups of researchers: one in charge of volumetric measures and the other in charge of planimetric measures.

Results:

The means of both methods were significantly different between AD and the other two diagnostic groups. In the differential diagnosis of AD against controls, 3D-yrA-H performed significantly better than 2D-yrA-MTL while differences were not statistically significant in the differential diagnosis of AD against MCI.

Conclusion:

Automated volumetry of the hippocampus is superior to manual planimetry of the MTL in the diagnosis of AD. Nevertheless, the 2D-yrAMTL is a simpler method that could be easily implemented in clinical practice when volumetry is not available.

## Introduction

Age-associated differences are detected in the medial temporal lobe (MTL) with an acceleration of atrophy starting around 72 years of age in healthy people [1-2]. In Alzheimer’s disease (AD), a disproportionate rate of atrophy in the medial temporal lobe (MTA) represents a reliable marker of the neurodegenerative process underlying clinical symptoms [3]. Then, age-associated changes in healthy people are modest, and the rate of atrophy over time is relatively slow, with a mean rate of tissue loss about 1% per year [1-2]. Accelerated MTA is a consistent finding in AD and mild cognitive impairment (MCI), with rates of about 2.5% in stable MCI and up to 4% in AD and MCI transitioning to AD (MCI progressors) [2, 4].

Numerous studies worldwide have shown that MRI is useful for detecting atrophy of structures in the MTL to discriminate early AD from controls and MCI, and have also demonstrated the potential for prediction of conversion from MCI to AD [5-12]. The European Union AddNeuroMed program and the US-based Alzheimer Disease Neuroimaging Initiative (ADNI) are two large multicenter initiatives designed to collect and validate biomarker data for AD that have allowed an impressive number of studies on brain atrophy in AD and MCI in the last decade. Both the AddNeuroMed and the ADNI cohorts showed very similar patterns of atrophy in MCI and AD [13].

The new diagnostic criteria for both AD and MCI due to AD incorporate MTA as a neuronal loss marker [14]. Thus, the time is now ripe for the quantification of MTL structures to become a component of the evaluation of patients with cognitive impairment.

Several methods have been proposed for quantifying atrophy of MTL structures using structural neuroimaging, including visual rating (VR), volumetry (3D), planimetry (2D), and linear measures (1D). In short, volumetry is based on the reconstruction of the volumes of regions of interest (ROIs), by integrating the area of brain structures across several MRI slices. Summed ROI over the slices is used to count the number of voxels and, ultimately, produce a volumetric measurement. The volumetric analysis provides an accurate and detailed measure of a predetermined ROI. For AD, the most commonly studied structure in the MTL is the whole hippocampus. While studies have established this clear finding of MTA in AD, hippocampal volumetry has not yet become a routine part of the diagnostic workup for neurodegenerative diseases. Despite the advantages, this method requires individual segmentation of structures within the MTL, which may also vary considerably across different operators, depending upon their training and experience. Once trained, segmentation of the hippocampus takes approximately 20–30 minutes, depending on the user’s experience, which limits its use in routine clinical practice [15]. An alternative is the use of automated MRI methods based on individual brain atlases that are able to capture signs of brain atrophy. FSL, SPM, and FreeSurfer packages are the most utilized methods in recent research for measuring brain atrophy. These packages allow for completely automated, user-independent calculations of regional brain volumes [16-19].

VR consists of semi-quantitative and subjective rating of ROIs, often on a single MRI slice. VR approaches are quick and can be assessed on large numbers of scans easily. Unfortunately, there is significant inter-observer variation [20-22]. Such poor inter-rater reliability is a major limitation for implementing VR in the clinical setting.

Linear methods (1D) use simple linear measures of the width or height of ROIs measured on one single MRI slice. Linear measures are objective and easy to obtain using any software for visualizing radiological images. A positive feature is that linear measures are available not only for MRI but for CT scans as well. Some studies have attempted to define sentinel changes that would allow the use of linear measurements of the hippocampus or the temporal horn to support clinical decision making. These studies have yielded variable results, with sensitivities ranging from 33% to 93% and specificity of up to 95% [23-25].

Planimetric measures are based on the area of ROIs measured on one single MRI slice. Several planimetric measures have proven to be easy to use in a clinical setting and to have good reproducibility [26-28]. Importantly, both the linear and the planimetric measures have the potential to be applied to computed tomography (CT) images, the most widely available imaging technique in primary health care, where most of the dementia evaluations take place [29]; it is often the first requested neuroimaging technique for routine assessment in many specialized centers.

An important limitation that obstructs the use of all these methods in clinical practice is the lack of normative values for interpretation. In addition, few studies have compared the performance of the different methods in the diagnosis of AD [30-33], and none of them included planimetric methods. Here, we aim to compare the results of one planimetric measure of the medial temporal lobes (the yearly rate of absolute atrophy of the MTL, 2D-yrA-MTL) with the results of automated volumetry of the hippocampi (the yearly rate of absolute atrophy of the hippocampus, 3D-yrA-H) in the differentiation of AD against controls and MCI patients.

## Materials and methods

### Patients and controls

A series of MRI studies on 290 subjects in the age range of 65-85 years, including patients with AD (n = 100), MCI (n = 100), and matched controls (n = 90) from the multi-center Pan-European AddNeuroMed study were included in this study [34].

All subjects underwent baseline and cognitive testing at baseline and every three months up to one year. The clinical assessment and cognitive testing of the AddNeuroMed subjects followed a standard protocol previously described [34]. Assessments included a structured interview including a detailed case and family history, the Cambridge Examination for Mental Disorders of Older People (CAMDEX), cognitive testing with mini-mental state examination (MMSE), Alzheimer Disease Assessment Scale – Cognitive (ADAS-cog), and stage of dementia with the Clinical Dementia Rating Scale sum of boxes (CDR-SOB) score. Written consent was obtained where the research participant had the capacity, and in those cases where there was dementia-compromised capacity, then assent from the patient and written consent from a relative, according to local law and process, was obtained. The detailed inclusion and exclusion criteria, as well as the diagnostic criteria, have been previously reported in other AddNeuroMed studies [34-36].

### Magnetic resonance imaging

Two 1.5T MRI studies were performed on all the patients, namely, baseline and follow-up (12 months) using the same machine and protocol. The imaging protocol included a high-resolution sagittal 3D T1-weighted magnetization-prepared rapid gradient-echo (MP-RAGE) volume and axial proton density/T2-weighted fast spin echo images.

### Neuroimaging analyses procedures

Researchers who participated in this study were divided into two independent subgroups, each subgroup blinded to the results of the other subgroup. The first subgroup of researchers performed volumetry of the hippocampus. First, we applied the FreeSurfer pipeline (version 4.5.0) to the MRI images to produce regional cortical thickness and subcortical volumetric measures [37-38]. All volumetric measures from each subject were normalized by the subject’s intracranial volume. Therefrom, we can compute the mean 3D-yrA-H using the following formulae: 3D-yrA-H = [(basal volume of both hippocampi) - (follow-up volume of both hippocampi)] x 1200 / [(months between MRI studies) x (basal volume of both hippocampi)]. Volumes from the left and the right hemisphere were averaged together. The second subgroup of researchers traced the areas needed to compute the 2D-yearly rate of atrophy of the medial temporal lobe (2D-yrA-MTL) as described previously [39]. Researchers used the free-hand tracing tool of the software Osirix to delineate the areas manually on one single coronal MRI slice passing through the interpeduncular fossa. The 2D-yrA-MTL consists of measuring the area of two brain regions on one single MRI coronal slide in T1-weighted sequence and then subtracting them. The two areas are (1) the MTL region (A) and (2) the parenchyma within the medial temporal region (B), including the hippocampus and the parahippocampal gyri (Figure 2). Therefrom, we can compute the mean 2D-yrA-MTL using the formulae described in the new terminology on regional brain atrophy [39]: 2D-yrA-MTL = [(basal A+A’–B+B’) - (follow-up A+A'–B+B’)] x 1200 / [(months between MRI studies) x (basal A+A’–B+B’)}. Areas of the left and the right hemisphere were averaged together.

### Statistics

As the main focus of the study was to compare two diagnostic procedures in AD, we did not differentiate MCI into converters and non-converters and used all MCI subjects as a single group. Statistical analyses were performed using the R™ and Epidata™ software. T-test analyses were used to test for differences in continuous outcomes, such as MRI-based measures and age between AD, MCI, and controls. The Chi-square test was used to test for differences in categorical variables, such as gender. We then set best cut-off points of these methods in the diagnosis of AD against controls and against MCI based on Youden's index and computed the resulting sensitivity and specificity. Finally, we produced receiver operating characteristic (ROC) curves of both 2D-MTA and hippocampus volumetry in the differential diagnosis of AD vs. controls and AD vs. MCI and compared them using the Hanley and McNeil method for comparing the ROC curves. 

## Results

### Demographics

Basic demographics and characteristics of the subjects in the different diagnostic groups are shown in Table 1.

**Table 1:** Demographics Basic demographics and characteristics of the subjects in the different diagnostic groups: first row shows the percentage of females and second row shows the mean age and standard deviation.

	Controls	MCI	Alzheimer’s
Gender (Female %)	53	49	64
Age in years	74.26 (5.22)	74.10 (5.12)	74.21 (6.19)

There were no statistically significant differences in gender (p = 0.242) and age (p = 0.458) between groups.

### Comparison of means between diagnostic groups 

The means of 3D-yrA-H and 2D-yrA-MTL in the three diagnostic groups are shown in Table 2.

**Table 2:** 3D-yrA-H and 2D-yrA-MTL Means and standard deviation of 3D-yrA-H and 2D-yrA-MTL in the three diagnostic groups.

	Controls	MCI	Alzheimer’s
3D-yrA-H	1.40% (0.25)	1.82% (0,23)	2.12% (0.20)
2D-yrA-MTL	1.31% (0.27)	1.79% (0.24)	2.24% (0.23)

Statistical analyses showed significant differences in all comparison of means of both methods between AD and the other two groups. Thus, the mean 3D-yrA-H was statistically different in AD compared with controls (p = 0.021) and MCI (p = 0.042), and the mean 2D-yrA-MTL was statistically different in AD compared with controls (p = 0.037) and MCI (p = 0.045).

### Performance in the diagnosis of Alzheimer’s disease

First, we set the best cut-off points for both methods in the diagnosis of AD versus controls and versus MCI based on Youden's index. Cut-off points are shown in Table 3. Table 3 also shows the resulting sensitivity and specificity values of each method using these cut-off points. 

**Table 3:** Cut-off Points Best cut-off points for both methods in the diagnosis of AD versus controls and versus MCI based on Youden's index, besides the resulting sensitivity and specificity values.

	Alzheimer’s vs Controls	Alzheimer’s vs MCI
3D-yrA-H	Cut-off: 1.67%; sensitivity: 86.8%; specificity: 82.1%	Cut-off: 1.92%; sensitivity: 76.7%; specificity: 73.1%
2D-yrA-MTL	Cut-off: 1.61%; sensitivity: 77.2%; specificity: 75.4%	Cut-off: 1.96%; sensitivity: 71.8%; specificity: 68.9%

**Figure 1:**
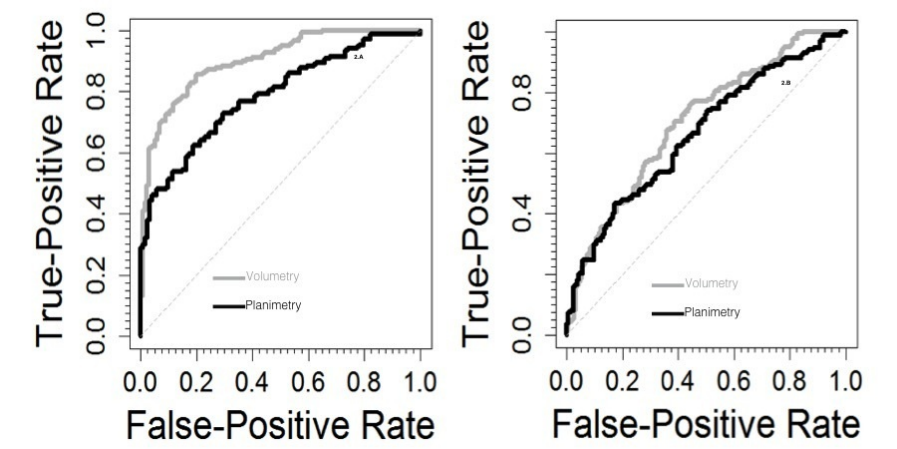
ROC Curves ROC curves in the differential diagnosis of AD vs controls (left) and AD vs MCI (right).

Then, we computed ROC curves and the area under the curve (AUC) for each method. In the differential diagnosis of AD against controls, the area under the curve (AUC) for 3D-yrA-H was 0.902 (0.87 - 0.93) while that for 2D-yrA-MTL was 0.785 (0.73 - 0.84); differences were statistically significant (p < 0.001) (Figure 1). In the differential diagnosis of AD against MCI, the AUC for 3D-yrA-H was 0.702 (0.65-0.75) while that for 2D-yrAMTL was 0.661 (0.60-0.72); differences were not statistically significant (p = 0.228) (Figure 1).

## Discussion

Despite the strong correlation to severity of AD pathology and inclusion in the new diagnostic criteria [14, 40], the measurement of MTA is not widely used in daily clinical practice as a criterion in the diagnosis of prodromal and probable AD. This is mainly because the methods available to date are sophisticated and difficult to implement in clinical practice (volumetric methods) or lack objectivity (VR). Planimetric methods, being objective and easy to implement, might be an alternative. However, no study has compared the performance of these methods against volumetric methods in the diagnosis of AD to date.

In this study, the means of both methods were significantly different between AD and the other two diagnostic groups. Rates of hippocampal atrophy were similar to rates reported in other studies, about 1.0-1.5%/year in healthy aging, above 1.5%/year in mild cognitive impairment, and around 2%/year in AD [2, 4].

In the differential diagnosis of AD from controls, 3D-yrA-H performed significantly better than 2D-yrA-MTL while differences were not statistically significant in the differential diagnosis of AD against MCI. Thus, 3D-yrA-H is superior to 2D-yrA-MTL in the diagnosis of AD.

Thus, we can conclude volumetric measures are more accurate in terms of sensitivity and specificity than planimetric measures in the diagnosis of AD. However, planimetric techniques have several advantages over volumetry from the clinician’s point of view. One of the strengths of planimetry methods is that they can be measured using almost any DICOM visualization software commonly used by clinicians or radiologists to visualize medical images worldwide. Most of these software packages are intuitive and require little or no training at all. Learning to trace the areas needed to compute the 2D-yrA-MTL is quick and easy, even for naive tracers [27]. Even more importantly, tracing these areas has good intra- and inter-rater reproducibility [27-28].

The main limitation of the 2D-yrA-MTL is that scoring is based on measurements performed on a single coronal slice, thereby providing a limited perspective of overall brain pathology. It is also expected that other conditions affecting the ventricular morphology, such as hydrocephalus, will probably alter the interpretation of the 2D-yrA-MTL. 

This study has some limitations. First, we have not taken into account the ApoE genotype of subjects, and therefore, we have not adjusted the analysis using this parameter (when ApoE genotype is a moderator of brain atrophy) [4]. As a result, there might be a bias in terms of a different distribution of ApoE genotypes between groups. Secondly, we are comparing a manual procedure for planimetric measures with an automatic procedure for volumetric measures. We know hippocampus atrophy rate estimates are greater when assessed with manual than with automatic segmentation, such as FreeSurfer [2]. Here, we are comparing results of manual procedures for planimetry measures with automatic procedures for volumetry measures when they do not estimate atrophy to the same extent. We also know these differences are related to the subregions included in each method rather than to the accuracy of estimation [2]. Finally, both methods used in this study are focused on the same region (the MTL), but the structures covered are largely different. The planimetric measures include the hippocampi and the parahippocampal gyri while the volumetric measures include the hippocampi only. Consequently, we assume from the start that the anatomical extension covered by these methods is not equivalent.

## Conclusions

Automated volumetry of the hippocampus is superior to the 2D-yrAMTL in the diagnosis of AD. Nevertheless, planimetry is a simpler method that could be easily implemented in clinical practice when volumetry is not available. 
